# Priapism as the first manifestation in chronic myeloid leukemia: A case report and focused review of literature

**DOI:** 10.1002/ccr3.4901

**Published:** 2021-11-19

**Authors:** Claudia Lucia Sossa Melo, Carlos Alberto Orozco Orozco, Angela Maria Peña Castellanos, Maria Alejandra Rueda Perea, Cristian Orlando Porras Bueno, Carlos Ivan Romero Diaz, Helga Natalia Rojas Rodríguez

**Affiliations:** ^1^ Servicio de Hemato‐oncología Fundación Oftalmológica de Santander Facultad Ciencias de la Salud Universidad Autónoma de Bucaramanga Bucaramanga Colombia; ^2^ Servicio de Hemato‐oncología Fundación Oftalmológica de Santander Programa para el Tratamiento y Estudio de Enfermedades Hematológicas y Oncológicas de Santander – PROTEHOS Bucaramanga Colombia; ^3^ Facultad Ciencias de la Salud Universidad Autónoma de Bucaramanga Bucaramanga Colombia

**Keywords:** BCR‐ABL positive, case reports, chronic, erectile dysfunction, leukemia, myelogenous, priapism

## Abstract

We report the case of a patient who was initially presented with ischemic priapism to the emergency department. He was treated with adrenaline intracavernous injections and aspiration with irrigation of the corpora cavernosa and distal shunt. In the postoperative period, anemia, basophilia, eosinophilia, thrombocytosis and hyperleukocytosis were detected. The patient was subsequently diagnosed with chronic myeloid leukemia.

Priapism is a rare manifestation of chronic myeloid leukemia (≤ 3%) and occurs mostly due to hyperleukocytosis, resulting in thrombus formation and corporal venous outflow obstruction. Priapism occurring in any setting is considered as a medical emergency that requires immediate local therapy because of resulting irreversible cell damage and fibrosis if not treated within the first 24–48 h.

## Graphical abstract

Priapism as the first manifestation of chronic myeloid leukemia is uncommon. However, given that delay in the treatment onset is associated with erectile dysfunction, urgent management is required.

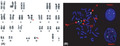

## 1 INTRODUCTION

Chronic myeloid leukemia (CML) is a clonal hematopoietic stem cell disorder characterized by a reciprocal translocation between the long arms of chromosomes 9 (ch 9) and 22 (ch 22). This results in the juxtaposition of the BCR gene from ch9 on ch22, encoding the BCR‐ABL1 gene, which is transcribed into mRNA and translated into the BCR‐ABL1 protein. The BCR‐ABL1 gene is always present in CML cases and provides a unique biomarker for diagnosis and monitoring response to the treatment. Furthermore, it acts as a susceptible drug target.^1^

The incidence of CML is consistent worldwide, at 1%–1.5 per 100,000 people. Despite the median age of onset of CML is 40–60 years, it may occur in children and the elderly population. It has a slight male predominance and has three stages: chronic phase (CP), accelerated phase (AP), and blast crisis (BC).^2^

CML patients typically present CP with leukocytosis, splenomegaly, fatigue, night sweats, malaise, weight loss, left upper quadrant pain, discomfort, and satiety. Other symptoms include lymphadenopathy, hepatomegaly, skin infiltration, bone pain, thrombosis and/or bleeding, retinal hemorrhages, extramedullary mass, and priapism. Priapism occurs due to hyperleukocytosis and subsequent hyperviscosity, causing thrombi, and venous obstruction.^1^, ^2^

Priapism is defined as an erectile penis for several hours in the absence of physical and psychological stimulation. Two types of priapism have been reported: ischemic or low‐flow priapism which represents approximately 80%–90% of cases and non‐ischemic or high‐flow priapism representing 10%–20% of cases.^3^

Non‐ischemic priapism usually occurs due to pelvic or genital trauma that leads to an arteriovenous fistula in the penile circulation. However, in ischemic priapism, sickle cell disease accounts for the majority of cases in the pediatric population, while in adults, it is often idiopathic. Besides, injection of vasoactive substances, such as papaverine and phentolamine, or other prescribed drugs for hematological diseases such as CML, polycythemia vera, and multiple myeloma can induce priapism.^3^, ^4^

Priapism is a urological emergency and a rare presenting feature of CML, requiring urgent treatment to prevent long‐term complications, particularly erectile dysfunction.^2^ Here, we report a case of a patient with CML who presented priapism as the first clinical manifestation.

## 2 CASE REPORT

A 47‐year‐old man, an afro‐descendant, consulted the emergency department due to an 8‐h sudden painful erection, without any recent sexual activity or trauma in the perineal region. He presented a history of arterial hypertension which was managed with losartan 50 mg/day and no other pathologies in his medical record. He denied the intake of additional medications. His vital signs were within the normal ranges at the first evaluation.

The urologists confirmed the case as ischemic priapism with a sudden onset with the penis exhibiting great turgidity resulting in intense pain. Given the risk of sexual impotence, adrenaline intracavernous injections were initiated every 10 min on three occasions along with intracavernous aspiration with irrigation without resolution. Upon treatment failure, a distal surgical shunt was made, achieving a painless partial erection with 60%–70% persistent penis turgency. A postoperative hemogram showed heterogeneous, normochromic, normocytic, moderate anemia with the presence of nucleated red blood cells, hyperleukocytosis, blasts, myelocytes, metamyelocytes, basophilia, eosinophilia, and thrombocytosis. Other laboratory tests revealed primary hypothyroidism and mild hyperuricemia (Table 1).

**TABLE 1:** Findings to laboratory test at admission.

Laboratory test	Parameter	Result	Reference Value
**Hemogram**	Hemoglobin	8,7 gr/ dl	14,0 – 17,5 gr/dl
	Hematocrit	26,7%	40 – 52%
	Leukocyte	221.930 /mm3	4.400 – 11.300 /mm3
	Lymphocyte	8.877 uI	1.500 – 4.000 uI
	Blast cells	15.535 uI	0
	Myelocytes	26.631 uI	0
	Metamyelocytes	4.438 uI	0
	Platelet	771000 /mm3	150.000 – 440.000 /mm3
**Other laboratories**	Uric acid	7,88 mg/dl	3,4 – 7,0 mg/dl
	Phosphorus	4,38 mg/dl	2,7 – 4,5 mg/dl
	Calcium	8.8 mg/dl	8,4 – 10,2 mg/dl
	TSH	7,63 uUI/ml	0,27 – 4,20 uUI/ml
	Free T4	0,833 ng/dl	0,9 – 1,53 ng/dl
**cRT‐PCR BCR/ABL1**	Evidence of bands associated with the p210 fusion protein of the BCR / ABL1 gene isoform b3a2 (446bp)		

The patient reported an unintended weight loss of 5 kg in the last 2 months and denied B symptoms, fatigue, or any other symptoms. On physical examination, only painful splenomegaly was observed. Considering the previous findings, CML was suspected and extensive examinations were performed. Bone marrow studies, reverse transcription‐polymerase chain reaction for Philadelphia chromosome, karyotype, and fluorescence *in situ* hybridization were performed.

**FIGURE 1:**
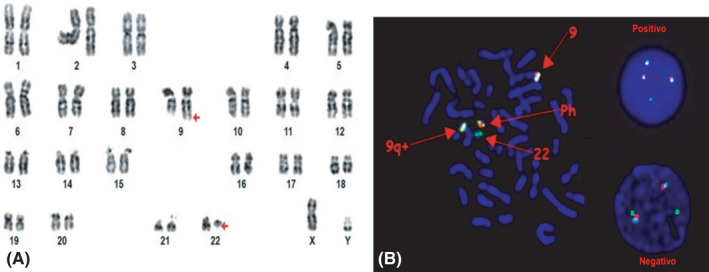
A, Karyotype analysis indicates the translocation t(9:22. [q34; q11.2]) (Arrows). B, Fluorescent in situ hybridization (FISH) display Philadelphia translocation.

Meanwhile, crystalloid fluid therapy, allopurinol, hydroxyurea and thyroid hormone replacement therapy were initiated. A decrease in the uric acid levels and leukocyte count were observed. Priapism resolved approximately 5 days after hospital admission. A positive BCR‐ABL1 gene and a bone marrow karyotype with abnormal translocation were observed (Figure 1). CML was diagnosed and a cytoreductive treatment with tyrosine kinase inhibitors was initiated with dasatinib at a daily dose of 100 mg. The patient was discharged with oral treatment and close follow‐up by a hematology service. At the 2‐month follow‐up, the patient had a complete hematologic response and a significant reduction in quantitative BCR‐ABL1; out of the 30 bone marrow cells analyzed in the metaphase, only two cells displayed 9:22 translocation. In the previous quantitative BCR‐ABL1, 28 out of 30 cells showed translocation.

## 3 DISCUSSION

CML patients usually present nonspecific symptoms such as night sweats, weight loss, fever, bone pain, abdominal pain, and fullness. However, some patients can exhibit symptoms related to leukostasis, such as thromboembolic phenomena, hearing loss, priapism, and neurologic deficits. Priapism as the first manifestation in hematological malignancies is rare, and CML represents about half of them.^4^

### 3.1 Epidemiology

Around 102 case reports have described priapism as manifestation of CML in a wide age range,^5^ some of them are listed below (Table 2). For example, Gupta et al. reported the phenomenon in a case of a 12‐year‐old child,^6^ while a similar incidence in older patients aged between 52 and 55 years have also been reported.^7^, ^8^ However, the youngest patient reported was 7 weeks old and the oldest 60 years old.^5^ Our patient was also in this age range.

**TABLE 2:** Some of the priapism and leukemia reported cases in the last 20 years.

Author (s)	Age	Diagnosis	Painful priapism (Y/N)	Duration of the complaint	First presentation (Y/N)	WCB cell/mm3	Signs and symptoms	Treatment
Gaye, Thiam et al. 2020 [9]	46 yrs	CML	Y	48 hrs	Y	526.000	Splenomegaly, hepatomegaly	Puncture of the corpora cavernosa with phenylephrine; hydroxiurea, imatinib
Dhar, Chhabra et al. 2019 [4]	52 yrs	CML	Y	4 hrs	Y	239.000	Pallor of the conjunctival mucosa, splenomegaly, hepatomegaly	Failed aspiration, Winter's procedure, hydroxyurea, imatinib
Qu, Lu et al. 2018 [3]	18 yrs	CML	Y	1 week	Y	257.000	Splenomegaly, hepatomegaly	Failed aspiration and puncture of the corpora cavernosa with phenylephrine, Winter's procedure; imatinib
Becerra, Jimenez et al. 2018 [6]	52 yrs	CML	Y	6 days	Y	282.000	Generalized fatigue and pallor, unintentional weight loss	Corpora cavernosa drainage‐irrigation and surgery penis shunts
Khan, Shafiq et al. 2018 [13]	16 yrs	CML	Y	11 days	Y	614.000	Splenomegaly, hepatomegaly, generalized pallor	Corporal irrigation and Winter shunting; hydroxyurea
Nerli, Magdum et al. 2016 [8]	19 yrs	CML	Y	24 hrs	N	296.800	Hepatomegaly, splenomegaly and pallor of the conjunctival mucosa	Puncture of the corpora cavernosa with phenylephrine; hydroxiurea, imatinib
Shaeer, Shaeer et al. 2015 [22]	21 yrs	CML	Y	6 days	Y	410.000	Splenomegaly, mild dyspepsia or sense of gastric fullness with meals	Failed aspiration and puncture of the corpora cavernosa with phenylephrine, penile prosthesis surgery; imatinib
Farhan, Anjum et al. 2015 [25]	38 yrs	CML	Y	30 hrs	Y	155.000	Pallor of the conjunctival mucosas, unintentional weight loss, dragging sensation in the abdomen and splenomegaly	Failed aspiration and puncture of the corpora cavernosa with phenylephrine; hydroxiurea, leukapheresis, imatinib
Gupta, Seth, Gupta. 2009 [5]	12 yrs	CML	Y	2 days	N	346.000	Splenomegaly, hepatomegaly, generalized pallor	Hydroxyurea, terbutaline, imatinib
Jameel and Mehmood. 2009 [7]	21 yrs	CML	Y	8 hrs	Y	316.000	Unintentional weight loss, epistaxis, pallor of the conjunctival mucosas, hepatomegaly, splenomegaly	Failed aspiration and puncture of the corpora cavernosa with phenylephrine; hydroxiurea
	55 yrs	CML	Y	12 hrs	Y	282.000	Pallor of the conjunctival mucosas	Aspiration; hydroxiurea
Ponniah, Brown and Taylor. 2004 [16]	19 yrs	CML	Y	18 hrs	Y	513.000	There were no other signs or symptoms	Failed aspiration and seven cycles of leukapheresis. Treatment for CML NA
Meng‐Wei et al. 2003 [23]	21 yrs	CML	Y	NA	Y	216.800	Unintentional weight loss, bleeding tendencies, hepatomegaly, splenomegaly	Failed aspiration and puncture of the corpora cavernosa with phenylephrine; hydroxiurea, interferon alfa−2a

Abbreviation: CML, Chronic Myeloid Leukemia; WBC, White blood cells; yrs, years; NA, not available; Y, yes; N, no.

### 3.2 Clinical Presentation

Several cases presenting a sudden painful erection due to ischemic priapism, as in our case, have been reported.^4^, ^9^, ^10^ Besides, subacute clinical presentations have also been described.^6^, ^7^, ^9^ Presentation of priapism in CML is almost always accompanied by splenomegaly and hepatomegaly^8^, ^9^, ^10^, ^11^, ^12^, ^13^, ^14^, ^15^; nevertheless, these clinical signs could be absent in some cases.^7^, ^16^, ^17^ In our case, only splenomegaly was observed.

### 3.3 Therapeutic approach

The American Urological Association (AUA) has developed an algorithm for the management of priapism, which provides guidelines for therapeutic options depending on the type of priapism.^18^ However, the safety and efficacy of different treatments are not well established, given that most evidence is derived from case reports and case series. Even so, ischemic priapism is considered a urologic emergency that must be resolved rapidly and appropriately to avoid permanent sexual dysfunction.^19^

The first line of treatment depends on the time of onset of priapism. If the duration is less than 4 h, intracavernosal injection of a sympathomimetic drug, preferably phenylephrine is recommended. However, if the duration is longer than 4 h, aspirations with or without irrigation must be added for optimal treatment.^18^ Phenylephrine is the preferred drug of choice because it has minimal cardiovascular side effects compared to other medications such as epinephrine and norepinephrine.^20^

If intracavernous aspiration and sympathomimetic therapy are ineffective, a surgical approach with a shunt between the corpus cavernosum and the corpus spongiosum, glans penis or one of the penile veins should be considered.^21^, ^22^ In our case, intracavernous injections with adrenaline plus aspiration with irrigation were initially performed because phenylephrine was not available; however, shunt surgery was required due to unsuccessful resolution.

For patients presented with priapism longer than 72 h, a penile prosthesis is suggested at the time of fistula surgery, given the poor prognosis of sexual function. Osama et al. described a case of priapism secondary to CML in a 21‐year‐old patient who required penile prosthesis.^23^

Other adjuvant non‐evidence‐based measures have been described in several case reports. For example, Gupta et al. described a case of a 12‐year‐old boy with intermittent 2‐week‐long priapism which later became sustained in the last 48 h and resolved after the CML management along with administration of subcutaneous terbutaline.^6^ Megan et al. described a case of a 20‐year‐old patient, where priapism was resolved only after localized penile radiotherapy following unsuccessful treatment with aspiration, phenylephrine injections, and CML management.^24^ However, Ergenc et al. described a case of an 18‐year‐old patient with priapism which was resolved only by initiating acute management of CML.^13^ Additionally, only one reported case treated with interferon α‐2a achieved adequate response with no recurrent priapism.^12^

Once CML diagnosis and hyperleukocytosis are made, a combined approach including hydration, systemic cytoreduction with hydroxyurea or tyrosine kinase inhibitors and therapeutic leukapheresis can be applied to reduce the white blood cell count and hyperviscosity.^25^, ^26^ It is important to educate the patient on therapy adherence, given that veno‐occlusive priapism can recur after abrupt discontinuation of treatment.^27^ Because leukemic priapism is a relatively rare incidence and the majority of recent literature includes small case series, there is no standard treatment recommended for leukemic priapism. However, the AUA strongly recommends that systemic treatment of an underlying disorder, such as CML, should not be undertaken as the only treatment for ischemic priapism.^18^ Intracavernous treatment is required and should be administered concurrently.

## 4 CONCLUSION

Incidence of priapism following CML is rare but can occur at any age. Physicians should consider it as a differential diagnosis, especially with evidence of hyperleukocytosis in a blood trial. Early management with systemic and local treatment provides satisfactory results and prevents long‐term sequelae.

## CONFLICTS OF INTEREST

None declared.

## AUTHOR CONTRIBUTIONS

Claudia Sossa, Carlos Orozco, and Angela Peña studied the conception and designed the case report and also reviewed the manuscript. Maria Rueda, Cristian Porras, Carlos Romero, and Helga Rojas, made the images, wrote and reviewed the manuscript. Also, they were involved in acquisition and analysis of data from the case report.

## ETHICAL APPROVAL

The manuscript is not being considered for publication elsewhere. Also, this work was published with the written consent of the patient.

## CONSENT

The authors confirm that during submission the patient consent has been signed and collected in accordance with the journal’s patient consent policy.

## Data Availability

Data openly available in a public repository that issues data‐sets with DOIs.
